# Methanol Extract of *Euchelus asper* Prevents Bone Resorption in Ovariectomised Mice Model

**DOI:** 10.1155/2014/348189

**Published:** 2014-06-05

**Authors:** Babita Balakrishnan, Shubhada Vivek Chiplunkar, Madhavi Manohar Indap

**Affiliations:** ^1^The Department of Zoology, The D.G. Ruparel College, Senapati Bapat Marg, Mahim, Mumbai 400016, India; ^2^Advanced Centre for Treatment Research and Education in Cancer (ACTREC), Tata Memorial Centre, Kharghar, Navi Mumbai 410210, India

## Abstract

Marine molluscs are widely distributed throughout the world and many bioactive compounds exhibiting antiviral, antitumor, antileukemic, and antibacterial activity have been reported worldwide. The present study was designed to investigate the beneficial effect of methanol extract of *Euchelus asper* (EAME) on estrogen deficiency induced osteoporosis in ovariectomised mice model. Forty-two female Swiss albino mice were randomly assigned into Sham operated (Sham) group and six ovariectomised (OVX) subgroups such as OVX with vehicle (OVX); OVX with estradiol (2 mg/kg/day); OVX with EAME of graded doses (25, 50, 100, and 200 mg/kg/day). Bone turnover markers like serum alkaline phosphatase (ALP), serum acid phosphatase (ACP), serum calcium, and histological investigations of tibia and uterus were analysed. Metaphyseal DNA content of the femur bone was also studied. Antiosteoclastogenic activity of EAME was examined. Administration of EAME was able to reduce the increased bone turnover markers in the ovariectomised mice. Histomorphometric analysis revealed an increase in bone trabeculation and restoration of trabecular separation by EAME treatment. Metaphyseal DNA content of the femur of the OVX mice was increased by EAME administration. EAME also showed a potent antiosteoclastogenic behaviour. Thus, the present study reveals that EAME was able to successfully reduce the estrogen deficiency induced bone loss.

## 1. Introduction

Osteoporosis is a condition of skeletal fragility characterised by decreased bone mass and microarchitectural deterioration of bone tissue, with a consequent increase in risk of fracture [1]. Osteoporosis is a worldwide health issue, with a high prevalence of disease not only in western countries but also in Asia and Latin America [2]. Women were found to have a higher risk of getting osteoporosis than men with the ratio of 1.6 : 1. Postmenopausal osteoporosis stems from the cessation of ovarian function at menopause which heightens and prolongs the rapid phase of bone loss characteristic of the early postmenopausal period [3]. It is associated with significant morbidity, mortality, reduction in quality of life, and increasing health care. About 1 in 3 women aged more than 50 years experienced an osteoporotic fracture in their lifetime. In women, osteoporosis and fractures mainly occur as a consequence of decrease in estrogen after menopause and result from an imbalance between bone resorption by osteoclasts and bone formation by osteoblasts, leading to a net bone loss with each remodelling cycle. Estrogen deficiency also augments erosion depth by extending the resorption phase of remodelling cycle through increased osteoclast life span due to its reduced apoptosis [4].

Estrogen replacement therapy (ERT) and combination hormone replacement therapy (CHRT) are two medication therapies often prescribed to supplement the decreased levels of hormones in postmenopausal women [5]. Research by Cauley et al. showed that hormone replacement therapy (HRT) can significantly improve bone mineral density (BMD) and reduce the risk for fractures of the vertebral spine, lower arm/waist and hip areas regardless of previous history, age, familial history, smoking, alcohol status, BMD, or fracture risk rates [6]. The Women's Health Initiative (WHI) suggests, however, that the long-term risks of HRT outweigh the benefits. It is associated with coronary heart disease, breast cancer, and stroke. In most countries, HRT is only recommended for climacteric symptoms, at a dose as small as possible and for a limited period of time. Thus, HRT is no longer recommended as a firstline treatment for the prevention and treatment of osteoporosis. Thus, the ultimate goal of treating women with postmenopausal osteoporosis is to identify compounds which not only reduce the risk of fracture but also have minimal side effects.

Despite the myriad of potential health benefits of the marine bioactives, there are few data relating to their possible preventive effect on osteoporosis. Efforts are needed to expand the marine based drug discovery to include osteoporosis disorders, which need extensive and urgent attention in terms of new therapies.

*Euchelus asper*, a marine mollusc that belongs to class Gastropoda, is a shell-oriented animal. It is commonly seen along the entire west coast and extends up to the south east coast in India [7]. It can be found on the rocky beaches near low tide marks. It is eaten by local people. Organic matrix from molluscan shells has the potential to regulate calcium carbonate deposition and crystallization. Biomineralization is one essential element in living organisms to protect themselves against predators and to form multicellular aggregates, for example, during bacterial biofilm formation, or in multicellular organisms, for example, in animals and plants [8]. Biomineralization is a highly orchestrated biological process in the mollusc. The organic matrix of the mollusc directs crystallization and the deposition and rate of crystals are also controlled by hormones produced by the mollusc [9]. Earlier studies by our group have shown that ether soluble fraction of* Euchelus asper* (EAE) exhibited immunosuppressive activity* in vivo* by plaque forming assay [10]. In the present study we have tried to investigate the antiosteoporotic effect of methanol extract of* Euchelus asper* (EAME).

## 2. Methods and Materials

### 2.1. Preparation of EAME and Its Fractionation

The organism exposed during the low tide was collected from the rocky shore of Khar Danda, Mumbai, Maharashtra, India. Itwas deshelled and the body mass was cold-percolated in methanol for 3-4 days. The resulting methanol extract was filtered and the residual animal mass was again immersed in fresh methanol. The process was repeated till the methanol extract became colorless. The filtered extracts were pooled and concentrated under reduced pressure and crude gummy methanol extract was obtained with a yield of 1.25%. It was refrigerated at −20°C till further use.

### 2.2. Animals and Treatments

Female Swiss albino mice of 18–22 g maintained at the Animal House of ACTREC were used for the study. This study was approved by Institutional Animal Ethics Committee (IAEC) of ACTREC, Tata Memorial Centre, Mumbai, India. They were fed with standard laboratory diet and water* ad libitum*. The mice were randomly divided into 7 groups, consisting of 6 animals in each group.

*Group A*. Sham control mice treated with vehicle (Sham control). 

*Group B*. Bilaterally ovariectomized control treated with vehicle (OVX). 

*Group C*. Bilaterally ovariectomized control treated with estradiol-2 mg/kg body weight (positive control). 

*Group D*. Bilaterally ovariectomized control treated with EAME 25 mg/kg body weight. 

*Group E*. Bilaterally ovariectomized control treated with EAME 50 mg/kg body weight. 

*Group F*. Bilaterally ovariectomized control treated with EAME 100 mg/kg body weight. 

*Group G*. Bilaterally ovariectomized control treated with EAME 200 mg/kg body weight.

All mice were bilaterally ovariectomized after anesthetizing with ketamine + xylazine. After 7 days of recovery from surgical convalescence, animals of groups D, E, F, and G were administered orally through stomach tube with EAME, 25, 50, 100, and 200 mg/kg body weight of EAME, respectively. The vehicle or extract was dissolved in sterilized water and was administered daily for 4 weeks. Animals in the control groups A and B received water (vehicle control) and of group C received estradiol (positive control). The body weight of the animals was recorded weekly during the experimental period.

### 2.3. Body Weight Measurements

On completion of the experimental period, the final body weights of animals of all the 7 groups were recorded. The animals were sacrificed under deep anaesthesia on the scheduled date and fresh weight of uteri was recorded.

### 2.4. Biochemical Estimations

#### 2.4.1. Estimation of Serum Calcium, Alkaline Phosphatase, and Acid Phosphatase

Blood was collected from retroorbital region and centrifuged at 1500 ×g for 20 min. Serum was separated immediately and was stored at −80°C. Serum alkaline phosphatase (ALP) and acid phosphatase activity (ACP) were estimated spectrophotometrically at 510 nm using Span Diagnostic Kit, Span Diagnostic Ltd., India. Serum was also analyzed for the presence of calcium. It was estimated by dry chemistry biochemical analyzer Vitros DT60II.

### 2.5. Histological Examination

#### 2.5.1. Tissue Collection and Processing

The tibia and uterus were harvested rapidly and fixed with Bouin's solution for 2 h, which was followed by decalcification in 5% nitric acid for tibia, dehydration in alcohol, clearing in xylene, and finally embedding in paraffin for further tissue section and histological staining [11].

### 2.6. Metaphyseal DNA Content

To measure bone DNA content, the metaphyseal tissues of the femur bone were shaken with 4.0 mL of ice-cold 0.1 M NaOH solution for 24 h after the homogenization of the bone tissues [12]. After alkaline extraction, the samples were centrifuged at 10000 ×g for 5 min and the supernatant was collected. DNA content in the supernatant was determined using the method of diphenylamine reaction and expressed as the amount of DNA mg/g of wet weight of bone tissue [13].

### 2.7. Cell Viability Analysis by MTT Assay

Spleens were removed from Swiss albino mice (18–22 g) under aseptic condition and splenocytes were isolated. Lymphocytes were isolated by Ficoll-Hypaque reagent. Mice splenocytes were plated in 96 well plates at a concentration of 1.5 × 10^5^ cells/well. EAME was dissolved in DMSO and dilutions prepared in RPMI-1640 supplemented with 10% FBS and cells were incubated for 72 hr. in 5% CO_2_ at 37°C. Control group received the same amount of DMSO. The general proliferation of lymphocytes was noted by reduction of the yellow dye 3-(4,5-dimethythiazol-2-yl)-2,5-diphenyl tetrazolium bromide (MTT) to form a blue product. After 4 hr. of incubation, the formazan product of MTT (Sigma-Aldrich, St. Louis, MO, USA) reduction was dissolved in DMSO, and absorbance was read using multiplate reader. The drug effect was quantified as percentage of control absorbance of the reduced dye at 540 nm. Cytotoxicity of EAME was checked within the concentration range of 0.97–500 *μ*g/mL [14].

### 2.8. *In Vitro* Osteoclastogenesis

Bone marrow cells were isolated from whole bone marrow of 4- to 6-week-old mice. Both the ends of bone of the femur were cut-off and the marrow cavity was flushed with *α*-Minimum Essential Medium (*α*-MEM, Sigma-Aldrich, St. Louis, MO, USA). The marrow cells obtained were washed with *α*-MEM and cultured in the same medium containing fetal bovine serum (FBS) at 1 × 10^7^ cells/mL in 24 well plates, in the presence of 10 ng/mL M-CSF at 37°C in 5% CO_2_. After 24 h, the nonadherent cells were further cultured on coverslips in a 35 mm culture dishes at a concentration of 1 × 10^6^ cells/mL and incubated with 30 ng/mL Macrophage-Colony Stimulating Factor (M-CSF) (Sigma-Aldrich, St. Louis, MO, USA) and 30 ng/mL receptor activator of nuclear factor kappa-B ligand (RANKL) (Sigma-Aldrich, St. Louis, MO, USA). One half of the old medium was replaced with fresh medium on third and sixth day. Extract was added to the culture medium at the beginning of the culture and at the time of medium change at various concentrations from 200 *μ*g/mL to 12.5 *μ*g/mL. The cells were cultured for 7 days [15].

After 7 days in culture, adherent cells were fixed and TRAP staining was performed using TRAP solution [16]. Briefly, cells were fixed with citrate buffered acetone. The fixed cells were then incubated for 1 hr. at room temperature in acetate buffer containing 10 mM sodium tartrate and naphthol AS-MX phosphate (Sigma-Aldrich, St. Louis, MO, USA) as a substrate and fast red violet LB salt (Sigma-Aldrich, St. Louis, MO, USA) as a stain for the reaction product. TRAP (+) multinucleated cells containing three or more nuclei were counted as osteoclast-like cells.

### 2.9. Statistical Analysis

Data are presented as mean ± SEM and analyzed by Student's* t*-test or one-way ANOVA followed by Tukey's test. *P* values less than 0.05 were considered statistically significant.

## 3. Results

### 3.1. Body Weight Measurements

After 4 weeks of treatment final body weights of all groups were not significantly different between the groups (data not shown).

OVX caused atrophy of the uterine tissue compared to Sham group. This uterine atrophy was caused by the depletion of the estrogen supplied by the ovary, thus indicating that the ovaries had been completely removed by the surgical procedure. This atrophy of uterine tissue was reflected in their weights. The ovariectomy-induced atrophy of the uterus was partially prevented by the estradiol treatment. EAME at the doses of 200, 100, and 50 mg/kg elicited uterotrophic effect by reducing the atrophy of the uterus induced by ovariectomy (Table 1).

### 3.2. Biochemical Estimations

#### 3.2.1. Estimation of Serum Calcium, Alkaline Phosphatase, and Acid Phosphatase

OVX mice had a high release of serum calcium in the blood as compared to Sham group (*P* < 0.01). This release of calcium in OVX mice was suppressed by EAME 100, 50, and 25 mg/kg treatment with significance value of *P* < 0.01. EAME 200 mg/kg treated group showed an increase in serum calcium as compared to OVX group (Table 1).

**Figure 1:**
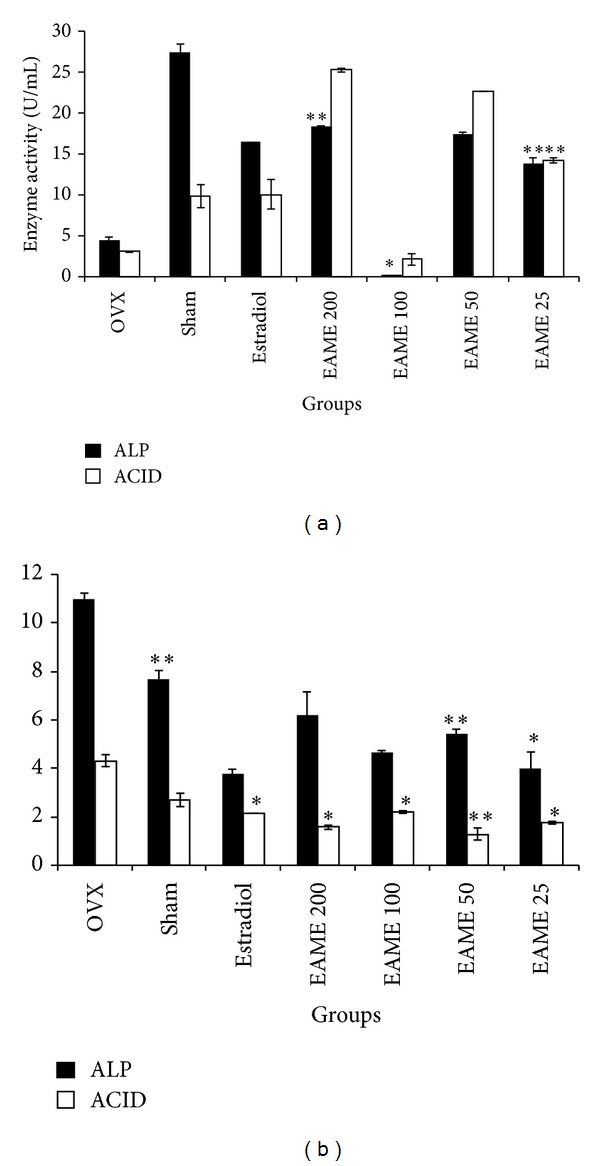
Effect of EAME on bone turnover markers alkaline phosphatase (ALP) and acid phosphatase (ACP). Serum was collected from control mice, ovariectomised mice treated with EAME and estradiol after the first and fourth week of oral administration of the extracts, and the levels of ALP, ACP were measured. (a) Effect of EAME on serum levels of ALP and ACP in OVX mice after the first week. (b) Effect of EAME on serum levels of ALP and ACP in OVX mice after the fourth week. All values are expressed as mean ± SEM, *P < 0.01 versus OVX, **P < 0.001 versus OVX.

ALP and ACP are bone formation and bone resorption markers, respectively, and can be estimated to investigate the bone turnover rate [17, 18]. ALP and ACP values were recorded after the first and fourth week of oral administration of EAME. ALP and ACP values after first week of oral administration were found to be random (Figure 1(a)). At the end of the experiment (after four weeks of oral administration), the ALP activity for OVX was higher than the Sham group (*P* < 0.001). Similarly OVX also increased the ACP activity in mice. Treatment with EAME 200, 100, 50 (*P* < 0.001), and 25 mg/kg (*P* < 0.01) could significantly reduce the increased ALP levels, whereas EAME 200 mg/kg (*P* < 0.01), 100 mg/kg (*P* < 0.01), 50 mg/kg (*P* < 0.001), and 25 mg/kg (*P* < 0.01) treated groups also revealed reduced ACP values as compared to OVX group. Estradiol treatment could also effectively decrease the elevated ALP and ACP activity (Figure 1(b)).

### 3.3. Histological Examination of Tibia and Uterus

**Figure 2:**
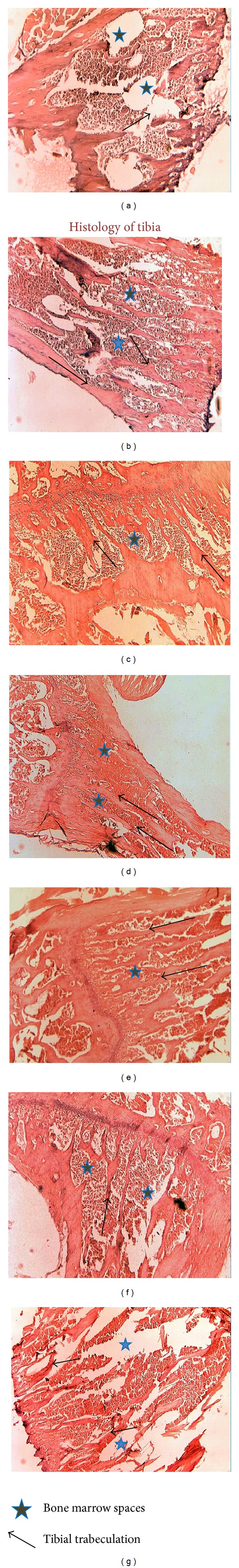
Histological study of the tibial trabeculation after the ovariectomy and EAME treatment. Ovariectomised mice were orally administered with EAME for 30 days. The tibia was harvested after the termination of administration of the extract. The tibia was formalin-fixed, decalcified, dehydrated in alcohol, fixed in paraffin, and Hematoxylin and Eosin stained (magnification 10x). (a) OVX control, (b) Sham control, (c) estradiol treated group, (d) EAME 200 mg/kg, (e) EAME 100 mg/kg, (f) EAME 50 mg/kg, and (g) EAME 25 mg/kg, where blue star indicates bone marrow spaces. Black arrow indicates tibial trabeculation.

The histological study of tibia revealed sparse, disrupted trabecular bone and empty bone marrow spaces in the OVX mice of the EAME treated group. Sham control mice showed good tibial trabeculation and filled bone marrow spaces. The restoration of the trabecular network with less intertrabecular spaces was observed in EAME 200 mg/kg, 100 mg/kg, and 50 mg/kg. Estradiol treated group also showed some improvement in the trabecular formation (Figure 2).

**Figure 3:**
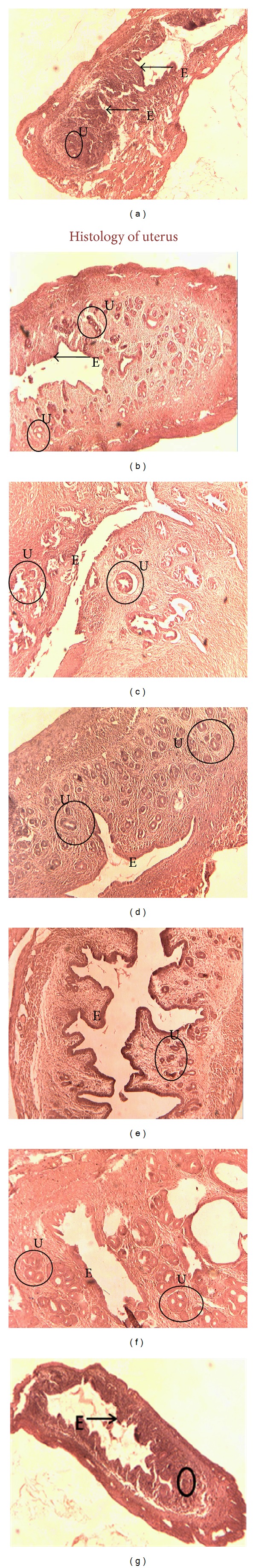
Histological study of the uterus after the ovariectomy and EAME treatment. Ovariectomised mice were orally administered with EAME for 30 days. The uterus was removed after the termination of administration of the extract. The uterus was formalin-fixed, dehydrated in alcohol, fixed in paraffin, and Hematoxylin and Eosin stained (magnification 10x). (a) OVX control, (b) Sham control, (c) estradiol treated group, (d) EAME 200 mg/kg, (e) EAME 100 mg/kg, (f) EAME 50 mg/kg, and (g) EAME 25 mg/kg. “U” (circled) indicates uterine glands; “E” indicates uterine epithelium.

OVX group showed many evident evaginations and hyperplasia of the epithelium was also observed. OVX group showed decrease in the number of uterine glands which appeared atrophied. EAME 200 mg/kg, EAME 100 mg/kg, and EAME 50 mg/kg inhibited the uterine epithelial distortion. The hyperplasia in the OVX control mice was reduced by EAME treatment. EAME treatment could maintain the intactness of the stromal tissue similar to Sham control group. In case of estradiol treated group, uterine glands were enlarged and atrophied and their epithelium showed distortion in the estradiol treated group. EAME treated groups showed no such distortion in the uterine glands similar to that of Sham control group (Figure 3).

### 3.4. Metaphyseal DNA Content

**Figure 4:**
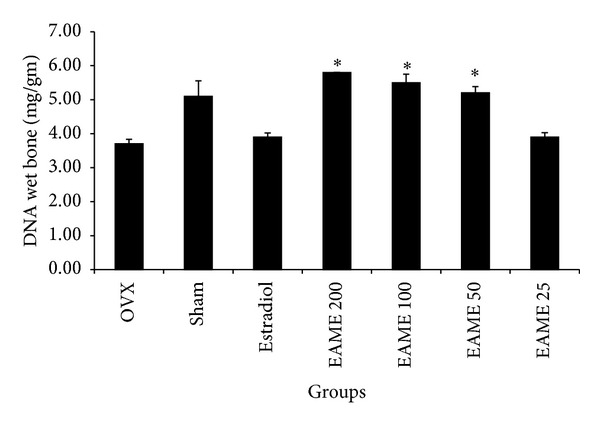
Effect of oral administration of EAME on DNA content of the metaphysis of the femur of ovariectomised mice. Mice were orally administered with the extracts for a period of 30 days. Animals were sacrificed after the termination of extract administration and femur was harvested. The metaphyseal tissue of the femur was obtained and homogenised in alkaline condition. The supernatant collected after centrifugation was measured for its DNA content. Each value is the mean ± SEM. *P < 0.05, compared with the OVX control value.

Bone growth can also be studied by investigating its DNA content which also reveals the number of bone cells including osteoclasts, osteoblasts, and osteocytes in bone cells [19]. The DNA content in the metaphyseal tissue was increased by EAME treatment (Figure 4). EAME 200 mg/kg (*P* < 0.05), 100 mg/kg (*P* < 0.05), and 50 mg/kg (*P* < 0.05) increased the metaphyseal DNA content as compared to OVX. Sham control also increased the metaphyseal DNA content but was not found to be significant.

### 3.5. Cell Viability Analysis by MTT Assay

**Figure 5:**
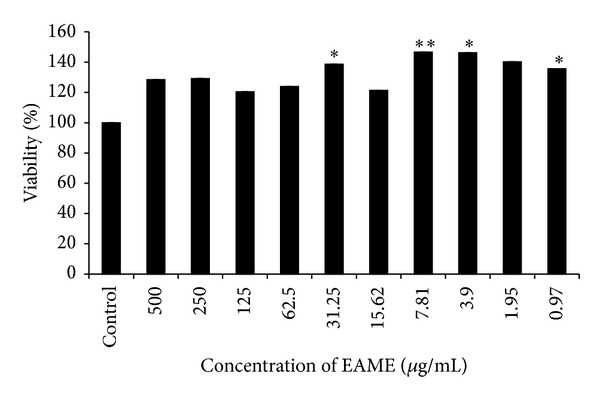
Effect of EAME on the viability of mice splenocytes. Viable cells as determined by MTT assay. Mice splenocytes (1.5 × 10^5^cells/well) were plated and cultured 72 hr. in the absence or presence of different concentration of extracts. Each bar represents mean ± SEM. *P < 0.05, compared with the control value.

In order to select the concentration for the bioassays, cytotoxicity of extract was determined on mice splenocytes. As shown in Figure 5, percentage survival of the cells treated with EAME was found to be more than the control revealing no cytotoxic effect of EAME on the cells. EAME did not show any cytotoxicity from the concentration range of 500 *μ*g/mL to 0.97 *μ*g/mL.

### 3.6. *In Vitro* Antiosteoclastogenesis

**Figure 6:**
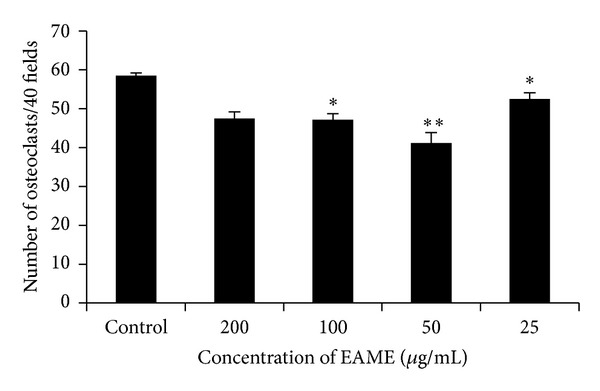
Antiosteoclastogenic effect of EAME. Bone marrow cells from 4–6 wk old Swiss mice were incubated on coverslips in 35 mm dish (1 × 10^7^ cells/mL) in the presence of M-CSF (30 ng/mL) and RANKL (30 ng/mL) with various concentrations of EAME (25 to 200 *μ*g/mL). After 7 d, cells were fixed and stained for TRAP, and the number of TRAP-positive MNC per well was scored. *P < 0.01, **P < 0.001 when compared to control, (*n* = 3).

Anticlastogenic data of EAME is presented in Figure 6 which shows that EAME suppressed the RANKL-mediated osteoclast formation significantly at concentration of 50 *μ*g/mL (*P* < 0.001) and 25 and 100 *μ*g/mL (*P* < 0.01).

## 4. Discussion

Postmenopausal osteoporosis is an estrogen-deficient state characterized by bone fragility, as the balance between bone resorption and bone formation shifts towards increased levels of bone resorption [20]. The safety of HRT is under question due to its compliance to serious side effects. Hence, it is plausible and logical to look for substances that have fewer side effects than estradiol in the hope of identifying some natural nutritional supplements that reduce menopausal symptoms and, perhaps, benefit bone without the adverse effects associated with HRT.

The ovariectomised (OVX) mice or rat models mimic changes in bone metabolism observed in postmenopausal osteoporosis and is considered as a gold standard for preclinical model for postmenopausal bone loss [21]. Ovariectomy in the mice results in an increase in bone turnover rate resulting in cancellous osteopenia [22]. Estradiol was used as the positive control in our study, because it is proved to have bone conserving effects and is endowed with good anabolic activities on bone* in vivo* [23].

In the present study we have attempted to investigate the effect of oral administration of the methanol extract of* Euchelus asper* on estrogen deficiency induced bone loss. The uterotrophic effect of estrogenic substances results in an increase in the uterine wet weight, which is an end point utilized in the standard uterotrophic assay [24]. A marked atrophy of the uterus has been used as evidence of the success of ovariectomy, because estrogen directly influences uterine weight [25]. The uterine weight which was decreased by ovariectomy was maintained by EAME supplementation as compared to Sham suggesting a uterotrophic activity of the compounds present in EAME. Bone acts as a reservoir for calcium and phosphorus. Bisphosphonates and vitamin D derivatives reduce the risk of hypercalcemia [26]. In our study, oral administration of EAME suppressed the loss of calcium in serum indicating its role in physiological calcium level.

Bone turnover markers are clinically reliable to predict imbalance between bone formation and bone resorption and, therefore, predict the rate of bone loss [27]. Alkaline phosphatase (ALP) is a key enzyme for bone calcification and provides an index of cell differentiation for bone formation [17]. Acid phosphatase (ACP), an important marker of bone resorption, positively correlated with histomorphometric indice of bone resorption. It is well known that estrogen decreases serum levels of these markers [18]. Serum concentration of both ALP and ACP was significantly greater in the ovariectomised control mice suggesting a high bone turnover rate, which were reduced by the administration of EAME. Postmenopausal bone loss is due to rapid bone metabolism in which bone remodeling is high. Menopause in women significantly increases bone resorption over formation due to low levels of estrogens thus inducing accelerated bone loss [28]. In the present study also ovariectomy has shown high remodeling by increase in bone turnover markers which was reduced by EAME supplementation.

Increased bone remodelling is associated with a transient loss of bone, increased porosity in the subchondral region, and reduced density [29]. The tibia of the OVX group of mice showed porosis and an empty bone marrow with less trabecular bone at the proximal head of tibia. However, remodeling lines were observed after administrating the ovariectomised mice with EAME for 4 weeks similar to the Sham control group. Histological assessment suggested that EAME induced the formation of new cancellous bone in the proximal tibial region to an equivalent extent as seen in the Sham control. Norzoanthamine, isolated from* Zoanthus* sp., a marine organism belonging to phylum Cnidaria, showed protective effects on both trabecular and cortical bone of the humerus in ovariectomised mice model. Norzoanthamine also accelerates the formation of a collagen-hydroxyapatite composite in bone which helps in bone formation, and hence it can be a promising drug candidate for osteoporosis treatment and prevention [30].

The histological assessment of uterine epithelium of the OVX group showed many evident evaginations and hyperplasia of the epithelium. EAME treated mice did not show any hyperplasia and evaginations of the epithelium. Also in the present study, estradiol treatment to ovariectomised mice exhibited endometrial hyperplasia. Similar results were reported by a previous study where treatment with ethinyl estradiol in ovariectomised mice produced significant increase in endometrial thickness, endometrial epithelial height, and the number of endometrial glands [31]. But EAME did not show any estrogenic effect as seen by estradiol treatment. Despite having similar effects on bone, the extract did not mimic the side effects of estrogen on reproductive tissues. Similar studies were conducted by Xie et al. for their plant extract of* Herba epimedii*, which also produced a beneficial effect on bone without inducing potentially harmful effect on reproductive organs [32].

DNA content in the bone tissues is an index of bone growth and the number of bone cells [19]. The intake of supplement containing zinc and the combination of *β*-cryptoxanthin (10^−9^ M) plus zinc (10^−6^ M) was found to cause a remarkable increase in alkaline phosphatase activity and DNA content in the diaphyseal and metaphyseal tissues in rats [33]. Yamaguchi et al. in their studies showed the synergestic effect of zinc with *β*-cryptoxanthin on bone components by studying its effect on metaphyseal DNA content [34]. In the present study, the DNA content in the metaphyseal tissue was increased by EAME treatment.

Bone resorption by activated osteoclasts with subsequent deposition of a new matrix by osteoblasts causes the formation of bone structure and bone remodeling. Imbalance between bone formation and bone resorption is the key pathophysiological event in many metabolic bone disorders in adult humans, including osteoporosis, a result of bone loss [35]. Thus it is accordingly thought that the medicines which improve the activity of osteoblast to facilitate osteogenesis and inhibit the activity of osteoclast to delay osteolysis are ideal therapeutic agents in osteoporosis.

The* in vivo* studies illustrated very well the antiosteoporotic effect of EAME. We further aimed to investigate whether this effect is reflected on the osteoclast by showing the antiosteoclastogenesis of the extract. The osteoclasts were cultured from mice bone marrow cells in the presence of RANKL, M-CSF, and the extracts. To discover novel inhibitors from EAME we did screening of osteoclast by staining TRAP activity which is involved in the bone resorption process of mature osteoclast. TRAP is considered a histochemical marker of osteoclasts and is highly expressed in osteoclasts in normal condition [36]. EAME significantly reduced the RANKL induced osteoclast formation. Bone erosion results from excessive osteoclast formation rather than damage to osteoblast activity. Therefore, the development of drugs that act to regulate osteoclast formation is important to prevent bone disease. Consistent with these results, Iejimalides obtained from marine tunicate* Eudistoma *cf.* rigida* which are unique 24 member-macrolides showed antiosteoporotic activity by inhibiting osteoclasts [37]. Our earlier investigation has shown that mollusc* Turbo brunneus *inhibits the bone resorption by acting on T cells to reduce the TNF*α* secretion which is responsible for osteoclast maturation and also revealed a potent antiosteoclastogenic activity [38].

In conclusion, the present study demonstrated that EAME could reduce the rapid bone loss occurring in ovariectomised mice leading to an imbalanced bone metabolism using bilaterally ovariectomised mice model. Our evidence clearly demonstrates that* Euchelus asper* could be considered as a natural alternative to hormone replacement therapy for the prevention and treatment of bone loss in postmenopausal women. Further research is required to elucidate and identify the bioactive compound present in EAME for its antiresorptive effect.

## Figures and Tables

**Table 1:** Effect of EAME on uterine weight and serum calcium of ovariectomised mice.

Parameters	OVX	Sham	Estradiol	EAME 200	EAME 100	EAME 50	EAME 25
Uterine weight (g/100bdywt)	0.12 ± 0.02	0.28 ± 0.02	0.36 ± 0.01	0.22 ± 0.00	0.2 ± 0.02	0.29 ± 0.05	0.04 ± 0.01
Serum calcium (mg/dL)	9.7 ± 0.066	8.8 ± 0.033*	9 ± 0.033	10.1 ± 0.185	9 ± 0.033*	9 ± 0.05*	8.6 ± 0.133*

Data presented as mean ± SEM, *P < 0.01 compared with OVX control value.
